# Therapeutic interventions for PTSD – current evidence on the the role of psychedelics

**DOI:** 10.1192/j.eurpsy.2021.1209

**Published:** 2021-08-13

**Authors:** I. Figueiredo, F. Viegas, F. Ferreira, A. Santos, J. Ramos, J. Miranda

**Affiliations:** 1 Mental Health Department, Hospital Professor Doutor Fernando Fonseca, Amadora, Portugal; 2 Psychiatry, Hospital Professor Doutor Fernando Fonseca, Lisboa (Amadora), Portugal; 3 Serviço Psiquiatria E Saúde Mental, Centro Hospitalar de Leiria, Amarante, Portugal

**Keywords:** psychedelics, MDMA, Ketamine, Posttraumatic Stress Disorder

## Abstract

**Introduction:**

Post-traumatic stress disorder (PTSD) is often a chronic condition, despite the existence of evidence-based treatment options. Psychotherapy is the designated first line treatment for PTSD, although high rates of psychiatric and medical comorbidity are observed among patients who have undergone treatment. The psychoactive properties of psychedelics may be of particular interest within a substance-assisted psychotherapy approach, offering new treatment opportunities for this debilitating disorder.

**Objectives:**

Review current evidence, therapeutic context, and possible mechanisms of action of different types of psychedelics in the treatment of PTSD.

**Methods:**

Literature review using Medline database.

**Results:**

3,4-methylenedioxymethamphetamine (MDMA)-assisted psychotherapy appears to be a potentially safe, effective, and durable treatment for individuals with treatment-refractory PTSD. Based on a small number of studies, ketamine administration appears to result in temporary symptom relief and may, in combination with psychotherapy, lead to lasting reductions in PTSD symptoms. Although these have not yet been investigated in controlled studies, it is known that psilocybin and LSD induce psychoactive effects that could as well contribute to the psychotherapeutic treatment of PTSD.

**Conclusions:**

The use of psychedelic compounds within a substance-assisted psychotherapy framework offers a novel method for pharmacotherapy-psychotherapy integration, although there is still much to learn from both a clinical and neurobiological perspective. It is necessary to generate more data regarding the safety and efficacy of psychedelics, in addition to research on cost-effectiveness, its use in mental health care infrastructure and also regarding the training of specialized therapists.

